# Endophytic microbe *Acinetobacter lwoffii* MA1 alters the cadmium content in *Spinacia oleracea* through membrane proteins-mediated adsorption

**DOI:** 10.3389/fmicb.2026.1922017

**Published:** 2026-09-16

**Authors:** Yu Zhang, Wei Chen, Qiming Tian, Junjun Zhou, Yachun Chen, Rujin Bai, Yiling Tian, Junfei Zhou, Lun Li, Jian Tian, Lei Zheng, Yingmin Jia, Aijin Ma

**Affiliations:** 1 School of Food and Health, Beijing Technology and Business University, Beijing, China; 2 College of Food Science and Technology, Hebei Agricultural University, Baoding, Hebei, China; 3 College of Life Science, JiangHan University, Wuhan, China; 4 State Key Laboratory of Animal Nutrition and Feeding, Institute of Animal Sciences, Chinese Academy of Agricultural Sciences, Beijing, China; 5 School of Food and Biological Engineering, Hefei University of Technology, Hefei, China

**Keywords:** *Acinetobacterlwoffii* MA1, Cd adsorption, endophytic microorganisms, membrane proteins, spinach

## Abstract

Vegetables such as spinach, celery, water spinach, and edible mushrooms are more prone to heavy metal accumulation. While the mechanism of heavy metal accumulation in vegetables and whether it is related to endophytic microorganisms remain unclear. In this study, we isolated an endophytic microorganism, *A. lwoffii* MA1, from spinach seeds that is tolerant to cadmium. Pot experiments demonstrated that enriching seeds with *A. lwoffii* MA1 increased the Cd content in spinach from 0.82 to 0.94 mg/kg, while microbial sequencing counts rose from 139.77 to 247.82, indicating that the addition of *A. lwoffii* MA1 affects Cd levels in spinach. Inoculation with *A. lwoffii* MA1 enhanced the microbial diversity of the spinach rhizosphere soil compared to the non-inoculated control, suggesting that the introduction of this endophytic strain positively modulates the indigenous soil microbial community. Subsequent analyses of the adsorption mechanism using SEM, ATR-FTIR, affinity chromatography, and mass spectrometry revealed that *A. lwoffii* MA1 captures positively charged Cd^2+^ through negatively charged cell surface groups such as amino and carboxyl groups. Additionally, three membrane proteins in the cell membrane may also play important roles in the adsorption process, including the TonB-dependent receptor, the RND transporter AdeIJK, and the outer membrane protein assembly factor BamD. These results provide novel insights into the influence of endophytic microorganisms on cadmium accumulation, offering a promising approach for mitigating cadmium content in spinach and other vegetables.

## 1 Introduction

Due to the extensive use of chemical fertilizers and pesticides, along with pollution from industrial wastewater, mining activities, and atmospheric deposition of heavy metals, the problem of heavy metal soil contamination in China has worsened significantly (Sari et al., 2022). Cadmium (Cd) is a toxic heavy metal characterized by strong mobility and lack of essentiality for humans and animals (Khan et al., 2017). The environmental pollution and ecological imbalance caused by Cd are becoming increasingly serious, attracting wide attention worldwide (Shi et al., 2022). The presence of Cd in soil damages the farmland ecosystems accumulates in plants, thereby threatening food safety and ultimately endangering human health. Cd exhibits high mobility within soil–plant systems, and even low concentrations can cause damage to plants and microorganisms (Shadmani et al., 2021). In fact, Cd contamination of the food chain begins with the its transfer from soil to plants. The primary source of Cd exposure for humans is through its uptake via plant-based nutrition. In living organisms, Cd tends to accumulate in different tissues and is distributed throughout plant roots, stems, leaves, flowers, fruits, and seeds. Plants that absorb excessive Cd suffer from serious negative effects, including inhibition of metabolism, oxidative stress, dysregulation of nutrient adsorption, and even death (Huang et al., 2018).

Spinach (*Spinacia oleracea*), a leafy vegetable characterized by broad green leaves, a rapid growth rate, and a high metal-accumulation capacity (Alia et al., 2015), is susceptible to Cd contamination. Some studies have reported that spinach accumulates and transports heavy metals from the roots to the stems and leaves, thereby increasing the risk of contamination within the chain (Verma et al., 2007). The contamination of water sources or soils in some areas with the heavy metal Cd has led to increased Cd levels in vegetables, exceeding the food safety standards (Liu et al., 2016). Excessive dietary intake of Cd-contaminated food can result in harmful accumulation, seriously impacting consumer health.

Endophytic bacteria refer to those living inside various tissues and organs of healthy plants without causing any visible symptoms. These bacteria provide greater protection to their host plants against both abiotic and biotic stresses compared to root-associated and foliar bacteria (Hardoim et al., 2015). The interaction between plants and endophytic bacteria significantly enhances the accumulation and mobilization of metals, thereby improving the plants’ resistance to metal-induced stress (Rattanapolsan et al., 2021). The adsorption and translocation of Cd by rice have been reported to be closely related to its endophytic microbial community, which influences Cd accumulation in rice plants (Qian et al., 2022; Tian et al., 2022). Moreover, heavy metal-tolerant endophytes have been detected in various plant parts, including leaves, stems, flowers, seeds, fruits, and roots. Common genera include *Bacillus*, *Pseudomonas*, *Stenotrophomonas*, and *Acinetobacter* (Compant et al., 2011). Endophytes are crucial symbionts that affect the host plant’s interactions with both abiotic and biotic environmental factors (Luo et al., 2019). In addition, plant seeds contain large numbers of endophytic and epiphytic bacterial communities, whose composition and vertical dispersal may directly or indirectly influence seed germination, plant growth, and health, potentially serving as a source of endophytes for the next generation of host plants (Du et al., 2021). Plant endophytes have been demonstrated to play a key role in the detoxification and transport of heavy metals within plants (Patel et al., 2018). Plant microbiota play important roles in host health, including protection against pathogenic bacteria (Tang et al., 2017), regulation of host gene expression, nutrient adsorption, and provision of metabolic capacity (Ramírez-Puebla Shamayim et al., 2013).

Plants have been plagued by heavy metal pollution for a long time, but how to effectively reduce the Cd content in vegetables through the plants themselves remains unclear. Endophytes, which are present in all plant species investigated to date, play an important role in plant health and development. This fact signifies that endophytic microorganisms in plants may affect the Cd content. Hence, in this study, we (1) screened, isolated, and identified Cd-adsorbing endophytic microorganism strains from spinach seeds; (2) conducted a pot experiment using these endophytic microorganisms to evaluate their effect on Cd adsorption in spinach; (3) investigated the mechanism of Cd adsorption by these endophytic microorganisms. The results aim to provide a foundation for future research on utilizing endophytic bacteria for heavy metal detoxification in crops, thereby paving the way for the development of sustainable agricultural practices.

## 2 Materials and methods

### 2.1 Isolation of spinach seeds endophytic bacteria

Select commercially available spinach seeds (OULAND, Henan, China). Initially, wash the spinach seeds three times with deionized water to remove all impurities. The washed seeds should first be immersed in 75% ethanol for 10 min, followed by immersion in a fresh sodium hypochlorite solution containing 10% available Cl^−^ for 10 min. Finally, immerse the spinach seeds in 75% ethanol for 3 min and wash them three times with sterilized deionized water. To confirm thorough surface sterilization, perform the last rinse with deionized water, then inoculate the seeds were inoculated onto LB agar plates. Subsequently, grind the treated seeds and place the homogenate in LB medium supplemented with Cd (20 mg/L), incubating the plates at 37 °C for 24 h. Transfer colonies exhibiting different morphologies to fresh LB plates to obtain pure isolates. Then, inoculate these isolates into LB liquid broth containing different concentrations of Cd (40 mg/L and 80 mg/L) to select for Cd-resistant strains. Purified isolates were then cultured in LB liquid broth and preserved at −80 °C in 25% (v/v) glycerol until further use. Reagents were purchased from Beijing Mirai Technology Co., Ltd.

### 2.2 Identification of Cd-resistant endophytic bacteria

The isolated bacteria were phenotypically characterized by observing colony morphology and gram staining results. Genomic DNA from each isolate was extracted using the TIAN amp Bacteria DNA Kit (TIANGEN Biotech, China), and the extracted DNA served as a template for amplifying the 16S rDNA. After assessing the quality and quantity via 1% agarose gel electrophoresis, Whole-genome sequencing of the bacterial isolates was performed by Allwegene Tech (Beijing, China). A Whole Genome Shotgun (WGS) strategy was employed, and libraries with varying insert sizes were constructed. These libraries underwent paired-end (PE) sequencing on the Illumina NovaSeq platform (Illumina, San Diego, CA, USA). Raw reads were quality-filtered and assembled using A5-MiSeq (Coil et al., 2015) and SPAdes (Bankevich et al., 2012) to generate draft genome sequences. The resulting sequences were analyzed using the nucleotide Basic Local Alignment Search Tool (BLASTn), and a phylogenetic tree was constructed using MEGA (Kumar et al., 2018).

### 2.3 Determination of Cd adsorption performance of endophytic bacteria

The living cells grown in LB medium were harvested by centrifugation and transferred into a Cd solution (40 mg/L, pH 6.0) at a final biomass dosage of 1.0 g/L (wet weight). The mixture was incubated at 37 °C and 200 rpm on an orbital shaker without addition of any nutrients. Then, 1% (v/v) of cells in the logarithmic growth phase were inoculated into LB medium supplemented with 40 mg/L Cd and cultured at 37 °C and 200 rpm for 48 h. After centrifuging the bacterial suspension (4 °C, 8000 rpm, 10 min), the supernatant was filtered, and the cells were collected in sterile saline. The cells were resuspended, rinsed by centrifugation, and then resuspended in 5 mL of sterile EDTA solution (0.5 mol/L). This suspension was vortexed at 1000 rpm for 1 min, centrifuged again, and resuspended in 5 mL of sterile 0.5 mol/L EDTA solution. The eluted supernatant was collected twice and the volume was adjusted to 25 mL. The cells rinsed before and after resuspension in the EDTA solution were freeze-dried. The dried samples were ground into powder and analyzed using an Agilent 720 inductively coupled plasma optical emission spectrometer (ICP-OES). The instrument was operated under the following conditions: radio frequency (RF) power of 1.20 kW, plasma gas flow rate of 15.0 L/min, auxiliary gas flow rate of 2.00 L/min, and nebulizer gas flow rate of 0.80 L/min. The sample uptake delay was set to 15 s, followed by an instrument stabilization delay of 30 s before data acquisition. Each sample was measured in triplicate, with a replicate read time of 15 s per replicate.

### 2.4 Pot experiment

The pot experiment was conducted in the greenhouse of Hebei Agricultural University. Briefly, the soil was air-dried and sieved to remove plant residuals, soil macrofauna, and stones. Three treatments were established, each replicated six times, using 6 kg (wet weight) of soil per pot and approximately 50 seeds per basin. In the first treatment group, seeds were allowed to grow normally (control group). In the second group, seeds were grow in soil containing Cd. In the third group, seeds were inoculated with beneficial microorganisms and grown in Cd-supplemented soil. CdCl_2_ was used to prepare 3 mg/L Cd concentration solution, and 1 L of this solution was added to each pot to achieve a soil Cd concentration of 0.5 mg/kg. The control group was supplemented with the same volume of tap water. Endophytic organisms were collected, and the suspension was adjusted with sterile saline to OD600 of approximately 0.8 (±0.01) to soak spinach seeds at a seed-to-solution ratio of s 1/10 (w/v, g/mL) at 25 °C and 80 rpm for 1 h. Seeds in the other two groups were soaked in sterile normal saline. The plants were cultured in a greenhouse at 25 °C with a 16:8 (day/night) photoperiod.

The soil was moistened with water and maintained at 60% of its water-holding capacity. The plants were harvested at 8 weeks after the inoculation treatments. Briefly, the plants were removed from their pots, and the soil was removed from the roots. The shoots and roots were separated and thoroughly washed to remove incidental soil and other adhering materials, ensuring minimal damage to the roots during rinsing. The plants were then rinsed thrice with deionized water to thoroughly remove the soil attached to the plant surfaces. Some randomly selected plant samples were used for DNA extraction. The remaining shoot samples were washed with deionized water and oven-dried at 80 °C for 48 h. The dried samples were ground into powder and analyzed by ICP for Cd quantification.

### 2.5 Multiple nucleotide polymorphism (MNP) analysis

The abundance of target strains was determined using multiple nucleotide polymorphism (MNP) markers following the MNP-Seq protocol (Fang et al., 2021). Genomic DNA was extracted from each sample using the TIANamp Bacteria DNA Kit. Multiplex PCR amplification was performed using a primer pool targeting pre-selected MNP marker loci specific to the target strain (Supplementary Table S1). PCR products were purified and then subjected to library construction, during which sample specific barcode sequences and sequencing adapters were appended to each amplicon library via a second round of PCR. The barcoded libraries were pooled in equimolar amounts and sequenced using paired-end sequencing on an Illumina NovaSeq platform with a read length of 150 bp. For data analysis, raw sequencing reads were quality filtered (Q > 20) and mapped to the reference genome using Bowtie2 (version 2.1.0) with default parameters (Langmead and Salzberg, 2012). For each MNP locus, an allele was defined as a haplotype of the SNPs within that locus. The allele supported by at least 20 mapped reads and with the highest read count was designated as the major allele. The abundance of the target strain was estimated based on the relative proportion of its characteristic MNP alleles among the total reads at each locus, calculated as the ratio of reads supporting the strain specific MNP allele to the total reads mapped to that locus. Samples were analyzed in technical triplicates to ensure reproducibility.

### 2.6 Analysis of soil microbial diversity

Soil genomic DNA was extracted using E.Z.N.A. Soil DNA Kit (Omega Bio-tek, Inc., USA) following the manual. The V3-4 hypervariable region of bacterial 16S rRNA gene were amplified withthe universal primer 338F (5′-ACTCCTACGGGAGGCAGCAG-3′) and 806R (5′-GGACTACNNGGGTATCTAAT-3′). The PCR products were purified using a Agencourt AMPure XP Kit (Beckman Coulter, Inc., USA). Sequencing libraries were generated using NEB Next Ultra II DNA Library Prep Kit (New England Biolabs, Inc., USA) following the manufacturer’s recommendations. The library quality was assessed by Nanodrop 2000 (ThermoFisher Scientific, Inc., USA), Agilent 2,100 Bioanalyzer (Agilent Technologies, Inc., USA), and ABI StepOnePlus Real Time PCR System (Applied Biosystems, Inc., USA), successively. Deep sequencing was performed on Illumina Miseq/Nextseq 2000/Novaseq 6,000 (Illumina, Inc., USA) platform at Beijing Allwegene Technology Co., Ltd. After the run, image analysis, base calling and error estimation were performed using Illumina Analysis Pipeline Version 2.6 (Illumina, Inc., USA). QIIME (Rognes et al., 2016) (v1.8.0) was used to generate rarefaction curves and to calculate the richness and diversity indices based on the OTU information., and use Graphpad pism9.5 software to plot. Based on the results of taxonomic annotation and relative abundance, Graphpad pism9.5 software was used for bar-plot diagram analysis.

### 2.7 Electron microscopy analysis

Cd-treated bacteria were collected at different time points and rinsed three times with phosphate-buffered solution. The samples were then dehydrated through a graded ethanol series, treated with a mixture of ethanol and isoamyl acetate for 30 min, and placed in pure isoamyl acetate overnight. The dehydrated samples were dried using a CPD300 critical point dryer (Ernst Leitz GmbH). After drying, the samples were mounted on conductive carbon tape, sputter-coated with gold (45 s, 10 mA), and examined using a Nova Nano 450 scanning electron microscope (FEI Company). Morphological imaging was performed with an SE2 detector at 3 kV, while Energy dispersive X-ray spectrometer (EDS) mapping was conducted at 15 kV to visualize elemental distribution.

### 2.8 ATR-FTIR analysis

The endophytic microorganisms were cultured in both normal and Cd-supplemented medium for 48 h. The two types of endophytic bacterial biomass were freeze-dried and ground into a fine powder. The powder samples were analyzed using a Thermo Fisher Scientific Nicolet iS50 FT-IR spectrometer equipped with a diamond attenuated total reflectance (ATR) module and a deuterated triglycine sulfate (DTGS) detector. Spectra were recorded over the wavenumber range of 400–4,000 cm^−1^ with a resolution of 4 cm^−1^ and 32 co-added scans per sample. Automatic atmospheric background correction was applied during data acquisition. Next, the amino, carboxyl, and phosphate groups on the bacterial surface were chemically masked, and their ability to adsorb Cd before and after blocking was measured. A formaldehyde-formic acid solution was used to mask the surface amino groups, a hydrochloric acid-methanol solution was used to mask the surface carboxyl groups, and a nitrilotriacetic acid-nitromethane solution was used to mask the surface phosphate groups. The corresponding Cd content was then detected by ICP, and the Cd adsorption rate was calculated. Finally, the bacterial cells were analyzed using ATR-FTIR.

### 2.9 Isolation, purification and identification of membrane proteins

Bacterial cells in the late-log phase were harvested by centrifugation at 8,000 × g for 15 min at 4 °C, resuspended in PBS to an OD600 of 1.0, and aliquoted into 20 mL portions. After centrifugation (8,000 × *g*, 10 min), the cell pellets were either stored at −80 °C or processed immediately. For protein extraction, the pellets were resuspended in 1 mL of extraction buffer containing 20 mM PBS (pH 7.4), 1 mM PMSF, and 10% (v/v) Triton X-114, followed by incubation at 4 °C for 2 h with gentle agitation. The lysate was centrifuged (8,000 × *g*, 5 min, 4 °C) to remove insoluble debris, and this step was repeated until no pellet was observed. The supernatant was then incubated at 37 °C for 30 min to and centrifuged at 3,000 × *g* for 10 min at room temperature to separate the organic phase from the aqueous phase. The organic phase was washed three times with PBS, and proteins were precipitated by adding nine volumes of ice-cold acetone and incubating at −20 °C overnight. The precipitated proteins were collected by centrifugation (13,000 × *g*, 10 min, 4 °C), resuspended in 100 μL of deionized water, and dialyzed against buffer for 24 h at 4 °C. The final protein sample was stored at −20 °C for further analysis.

The protein purification process was carried out using a Cd-charged affinity column. First, the column was treated with a stripping buffer containing 50 mM PBS (pH 7.0), 0.5 M NaCl, and 0.1–0.2 M EDTA to remove any bound nickel ions. Following EDTA treatment, the column was washed with 50 mM PBS containing 0.5 M NaCl to eliminate residual EDTA. The column was then charged with a 0.2 M solution of CdSO_4_ or CdCl_2_ to facilitate Cd^2+^ binding to the ligand. After Cd^2+^ binding, the column was thoroughly washed with 50 mM PBS to remove any unbound metal ions. Finally, the column was equilibrated sequentially elution buffer and equilibration buffer before use. The protein sample was loaded onto the Cd column three times to ensure efficient binding. Target proteins were eluted using a solution containing 0.1–0.2 M EDTA in 50 mM PBS. The eluted protein fractions were analyzed by SDS-PAGE, and the band corresponding to the target protein was excised for subsequent identification. The band of interest was protease-digested, desalted using ZipTip® C18 pipette tip (Merck KGaA, Darmstadt, Germany), and the peptides were detected by RPLC-MS (Thermo EASY nLC 1,000 Liquid Chromatography, Thermo Scientific QE Mass Spectrometer).

## 3 Results

### 3.1 Isolation and identification of the endophytic bacteria

A total of 10 culturable endophytic bacterial strains were isolated from spinach seeds based on differences in colony morphology. These 10 strains were inoculated onto plates and into liquid media containing 40 mg/L Cd^2+^. Six strains that exhibited better growth in the 40 mg/L Cd^2+^ medium were subsequently inoculated into a medium containing 80 mg/L Cd^2+^ and incubated. One strain was identified that grew well at 80 mg/L and demonstrated strong tolerance to Cd. Due to its high tolerance, this strain was designated MA1 for further analysis in this study. Based on the analysis of the 16S rDNA sequence, among the sequences published in the NCBI database, strains with high homology were screened. The results showed that the strain MA1 was the best match for *A. lwoffii* strain EK30A, with a similarity of 98.59%. Phylogenetic analysis further confirmed its placement within the *Acinetobacter* genus, clustering closely with *A. lwoffii* (Figure 1a). Therefore, we named it *Acinetobacter lwoffii* MA1 (*A. lwoffii* MA1).

**Figure 1:**
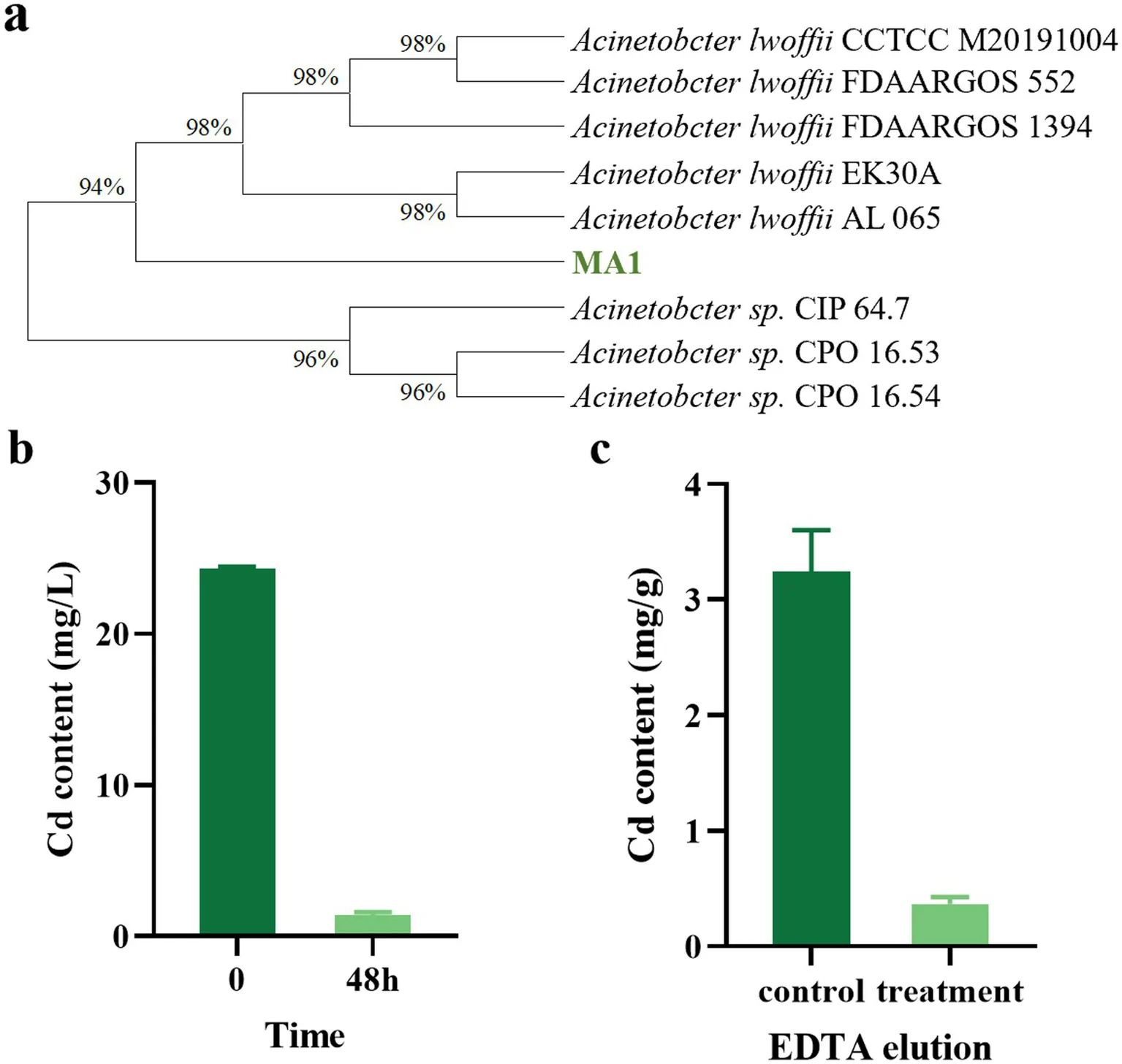
Characterization of strain *A. lwoffii* MA1. **(a)** Phylogenetic tree analysis of strain MA1 based on partial 16S rDNA gene sequence. **(b)** Cd content in the supernatant before and after 48 h. **(c)** Cd content in bacterial cells before and after EDTA elution.

### 3.2 Cd adsorption capacity of *A. lwoffii* MA1

The adsorption quantity and distribution of Cd^2+^ by *A. lwoffii* MA1, both intracellularly and extracellularly, at 40 mg/L Cd^2+^ were further determined. Notably, after 48 h of incubation, the Cd^2+^ concentration in the supernatant was only 1.378 mg/L, indicating an adsorption capacity of 94.36%by *A. lwoffii* MA1 (Figure 1b), which is higher than that reported for most other strains (Zhang et al., 2019). Additionally, the cellular localization of Cd^2+^ adsorption was examined in greater detail. The Cd^2+^ content in the cells before and after EDTA elution was 3.24 mg/g and 0.37 mg/g, respectively (Figure 1c). Remarkably, the results demonstrated that outer membrane proteins may contribute to Cd^2+^ sequestration by *A. lwoffii* MA1, with 88.58% of the adsorbed Cd detected in the outer membrane fraction. However, contributions from other cellular components cannot be excluded, and the relative contribution of each fraction requires further investigation. These findings indicate a potential metal resistance mechanism in this strain, although the molecular basis remains to be elucidated.

### 3.3 Endophytic microbial enrichment increased the Cd content of spinach

To assess the application of strain *A. lwoffii* MA1 as a facilitator for a Cd adsorption agent, we examined its effects on spinach seeds growth and its Cd adsorption capacity. The study measured Cd concentrations and *A. lwoffii* MA1 levels in spinach leaves under three conditions (control, Cd, and *A. lwoffii* MA1 + Cd). After a two-month cultivation period, spinach grown in Cd-supplemented soil exhibited poor growth, with leaves appearing curled and yellowed compared to the control group (Figures 2a–c). The results revealed that Cd exposure severely hindered growth. Further investigation of Cd levels in spinach leaves revealed that the spinach seeds inoculated with *A. lwoffii* MA1 could significantly increase Cd accumulation compared to untreated seeds. In soil supplemented with Cd, the inoculation with of *A. lwoffii* MA1 led to a dramatic rise in Cd content, resulting in a 14.5% increase in Cd adsorption. Specifically, the Cd content in spinach leaves increased from approximately 0.822 mg/kg to 0.941 mg/kg (Figure 2d). The *A. lwoffii* MA1 content in spinach leaves was further analyzed using the MNP assay, with relative content represented by the number of reads; a higher number of reads indicated a greater relative content. Further investigation revealed that spinach seeds enriched with *A. lwoffii* MA1 could significantly increase the bacterium’s content in spinach leaves compared to normal seeds. Compared with normal seeds grown in ordinary soil, the *A. lwoffii* MA1 content in spinach from seeds enriched with *A. lwoffii* MA1 increased from 69.473 to 247.823, representing a 256% increase (Figure 3). Similarly, compared with normal seeds grown in cadmium-contaminated soil, the *A. lwoffii* MA1 content in spinach from enriched seeds increased from 139.277 to 247.823, an increase of 77.9%. The above results demonstrate that enriching spinach seeds with *A. lwoffii* MA1 leads to a higher abundance of this bacterium in the leaves. Spinach is capable of adsorbing Cd in the soil through *A. lwoffii* MA1. Spinach seeds enriched with *A. lwoffii* MA1 will lead to spinach containing higher levels of both Cd and microorganisms. Spinach with low Cd content has a lower abundance of *A. lwoffii* MA1, while spinach with high Cd content has a higher abundance of this bacterium. There is a positive correlation between the content of endophytic bacteria in spinach and the Cd content. That is to say, the amount of Cd absorbed by the plants is influenced by the population of relevant endophytic bacteria.

**Figure 2:**
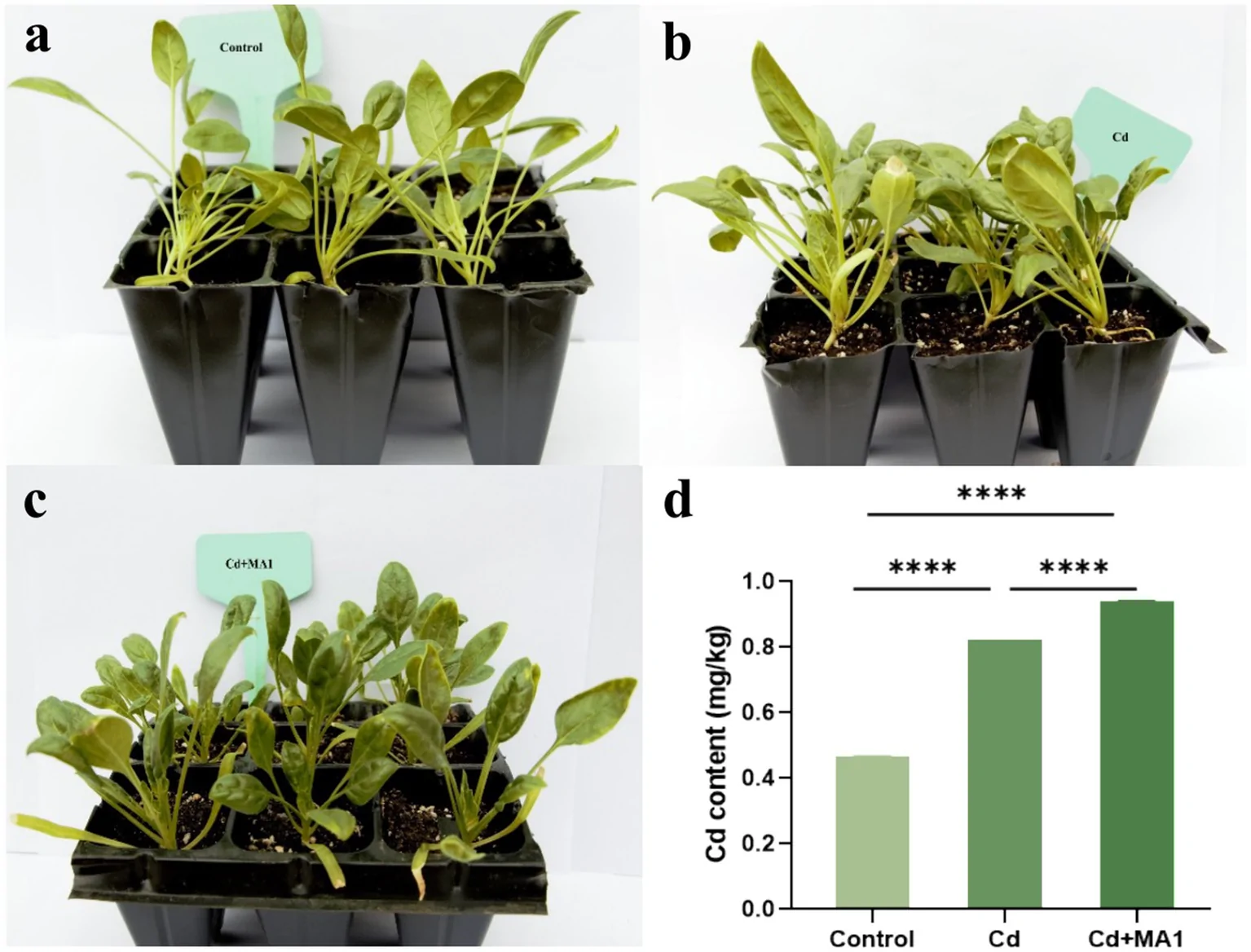
Impact of *A. lwoffii* MA1 inoculation on spinach. **(a–c)** Effects of *A. lwoffii* MA1 on spinach growth. **(d)** Cd content of spinach leaf. Control: Soil treated with sterile water and normal seeds; Cd: Soil treated with Cd and normal seeds; Cd + MA1: Soil treated with Cd and seeds reinforced *A. lwoffii* MA1. **** *p* < 0.0001.

**Figure 3:**
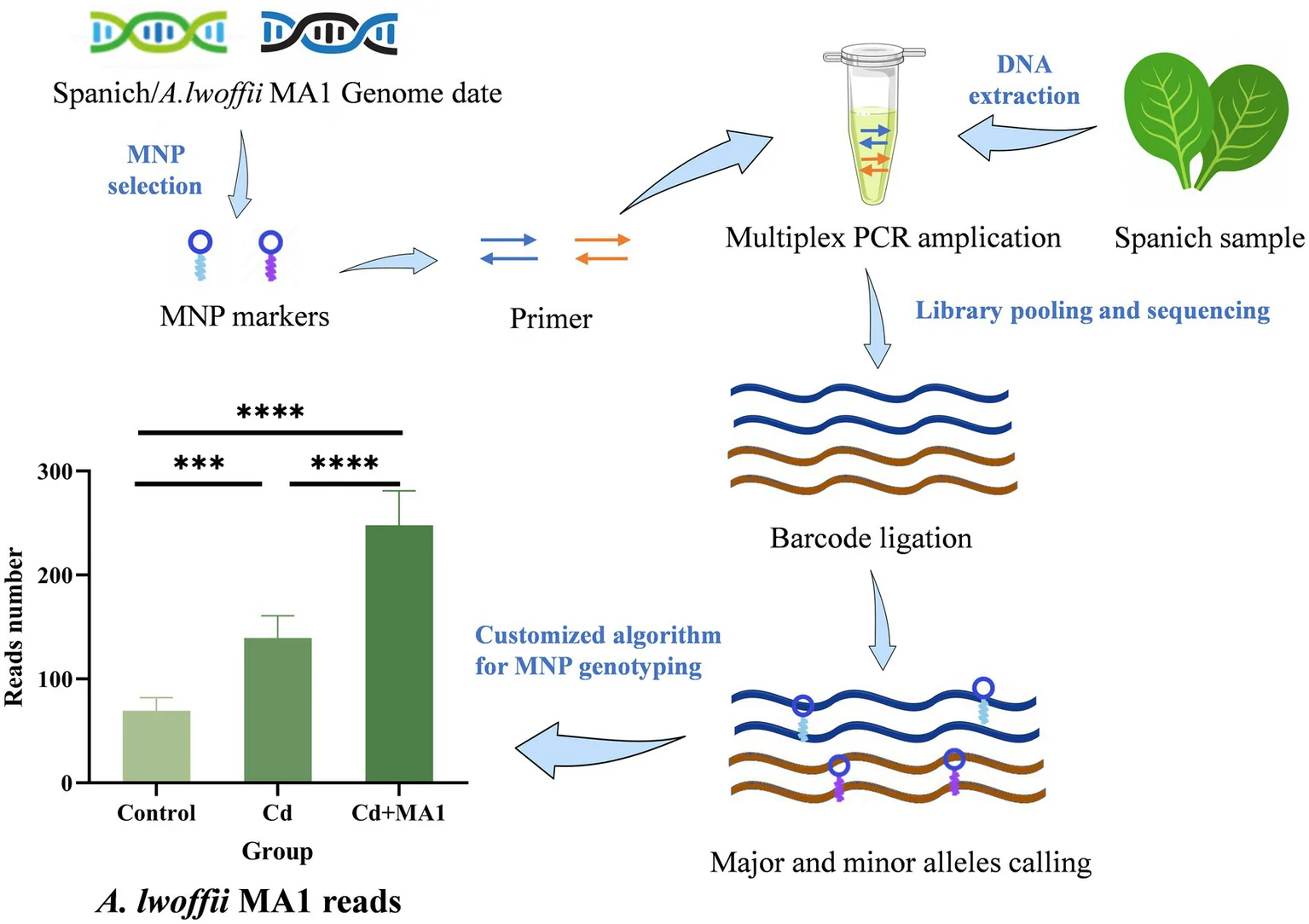
The relative content of *A. lwoffii* MA1 in spinach. ****p < 0.0001.

### 3.4 Bacterial community in rhizosphere soil of spinach

Analysis of soil bacterial community *α*-diversity revealed that the Goods coverage values in all three groups were 0.93, indicating that the sequencing depth was sufficient to cover the vast majority of bacterial species in each sample and that the data were reliable. In terms of species richness, the Chao1 and Observed species indices in the Cd treatment group were significantly lower than those in the CK group (Figure 4), suggesting that Cd contamination severely inhibited the species richness of the soil bacterial community. In contrast, both indices in the Cd + MA1 group showed a marked recovery compared to the Cd group, nearly returning to the levels observed in the CK group, indicating that the addition of *A. lwoffii* MA1 effectively alleviated the negative impact of Cd contamination on species richness. The PD whole tree exhibited a similar trend, suggesting that *A. lwoffii* MA1 supplementation partially restored the damage to community evolutionary lineage diversity caused by Cd stress, although the degree of recovery was less pronounced than that observed for species richness. The Shannon index decreased in both the Cd and Cd + MA1 groups, with little difference between the two, implying that *A. lwoffii* MA1 had a limited effect on improving overall community diversity and evenness. The Simpson index remained close to 0.99 across all three groups, indicating that the species distribution within the soil bacterial community was relatively even regardless of treatment, with no single dominant species being overly prominent. Collectively, these results suggest that Cd contamination primarily suppressed species richness and phylogenetic diversity of soil bacteria, and while *A. lwoffii* MA1 addition effectively restored species richness, its improvement of community evenness was not significant.

**Figure 4:**
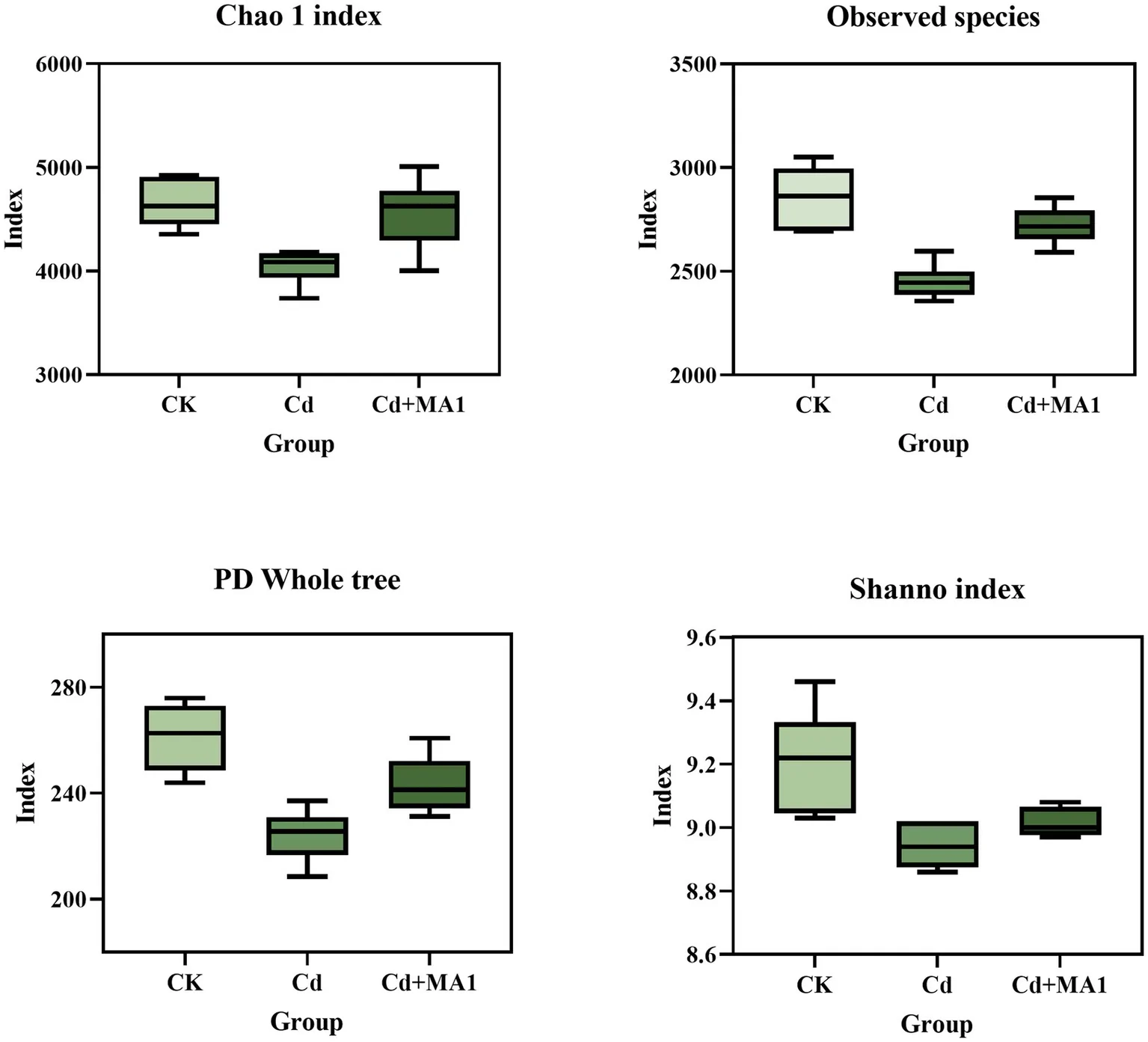
Alpha diversity index of spinach rhizosphere soil.

At the phylum level, the dominant bacterial phyla across the three groups were Bacteroidetes, Proteobacteria, and Actinobacteriota (Figure 5a). The community structure of the CK group was similar to that of the Cd + MA1 group, whereas the Cd group exhibited a distinctly different community composition, indicating that Cd contamination significantly altered the phylum-level taxonomic composition of the soil bacterial community, and that MA1 addition partially restored the community structure toward that of the CK group. At the genus level, differences in community composition were more finely resolved. Compared with the Cd group, the Cd + MA1 group showed a marked increase in the relative abundances of *Pricia*, *Actinomadura*, *Rhodanobacter*, *Lysobacter*, *Luteimonas*, *Salinimicrobium*, and *Arenibacter*, while the relative abundances of *Saccharimonadales*, *Bacillus*, and *Geobacillus* decreased (Figure 5b). These results indicate that MA1 treatment contributed to the restoration of multiple beneficial functional bacterial genera in Cd-contaminated soil, while simultaneously suppressing the overgrowth of certain tolerant or conditionally dominant bacteria. At the phylum level, *Acinetobacter* sp. was present in the soil samples. However, at the genus level, *A. lwoffii* was not identified. Rhizosphere microorganisms did not contain *A. lwoffii*, which was detected only in plant leaves as an endophytic microorganism of spinach. Large numbers of *A. lwoffii* are found exclusively in spinach leaves and not in soil. These results further indicate that endophytic microorganisms of plants influence Cd adsorption. The relationship between endophytic microorganisms and Cd content can be leveraged to reduce Cd pollution in plants.

**Figure 5:**
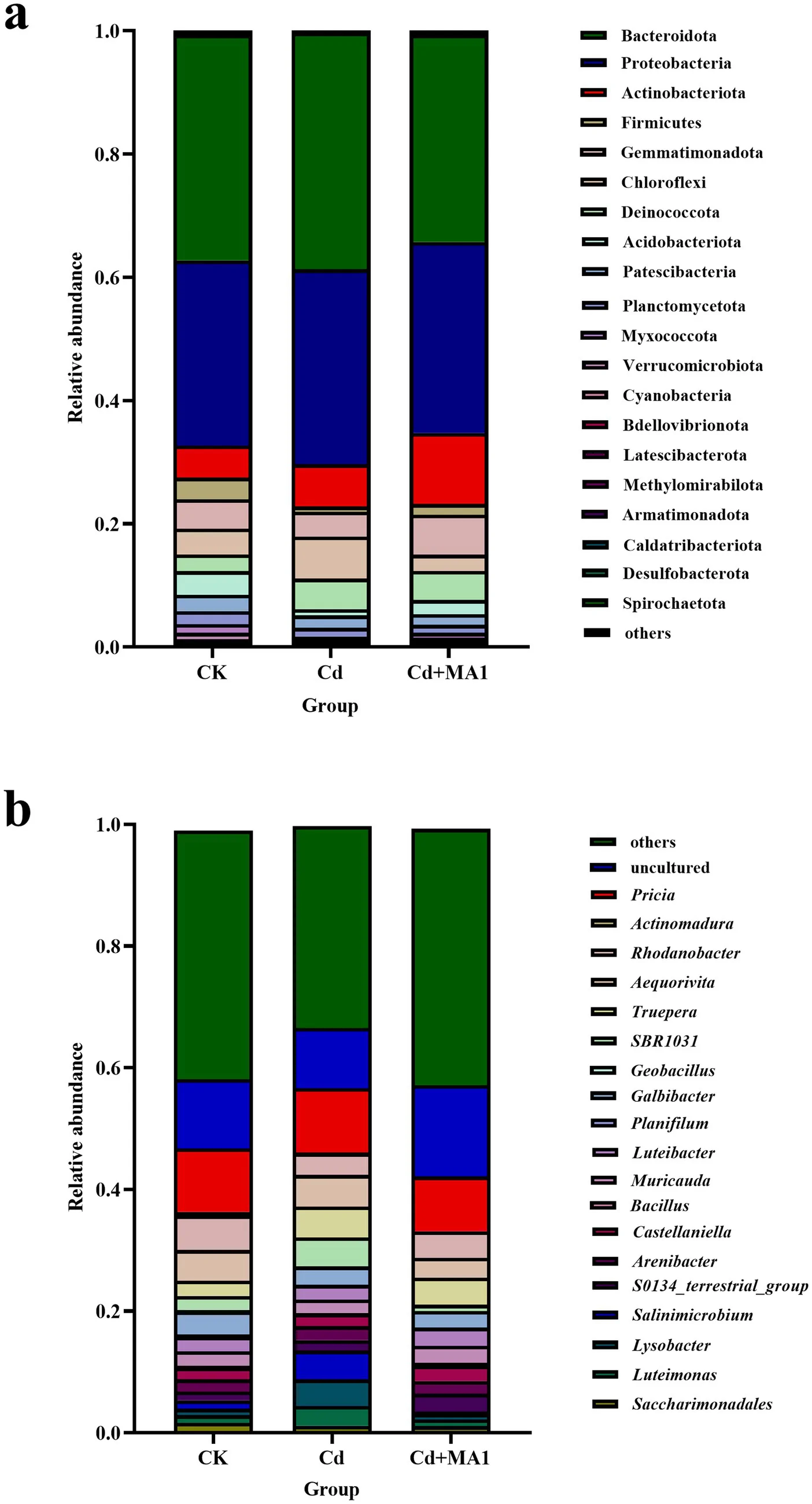
Microbial composition in spinach rhizosphere soil at the phylum **(a)** and genus **(b)** levels.

### 3.5 Characterization of Cd adsorption by *A. lwoffii* MA1

We characterized the time-dependent morphological changes of strain *A. lwoffii* MA1 under Cd^2+^ exposure to elucidate its Cd tolerance mechanisms. As shown in Figure 6a, the natural cells exhibited a smooth surface and a plump, short rod shape, somewhat resembling a spherical form. After exposure to 40 mg/L Cd^2+^, some depressions and wrinkling were observed, but no holes appeared on the cell surface (Figures 6b,c). In addition, after 2 days of Cd exposure, the presence of Cd, indicated by yellow dots, was detected within the cells (Figure 6d), indicating that Cd is almost uniformly distributed in the cytoplasm. Meanwhile, elemental analysis using EDS confirmed the binding of Cd^2+^ to the surfaces of *A. lwoffii* MA1 cells, detecting elements such as C, O, N, P, S, and Cd (Figure 6e).

**Figure 6:**
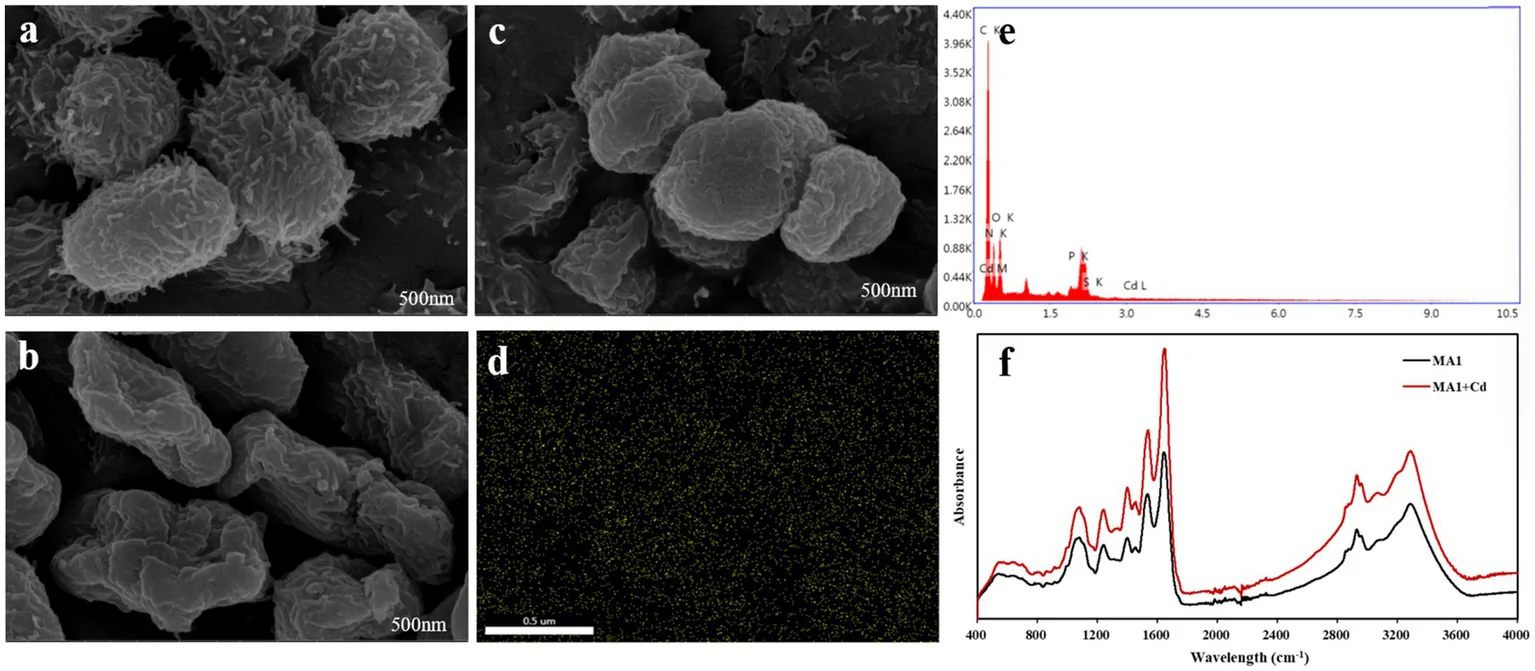
SEM analysis and ATR-FTIR spectroscopic analysis. **(a–c)** SEM images of *A. lwoffii* MA1 before and after exposure to Cd^2+^. **(d)** Mapping image of cell incubated with Cd for 2d. **(e)** EDS analysis verifying the presence of various elements on the cell surface of *A. lwoffii* MA1 exposed to Cd. **(f)** MA1: *A. lwoffii* MA1 was cultured in normal medium; MA1 + Cd: *A. lwoffii* MA1 was cultured 2d in medium added Cd.

To further investigate the Cd-binding capacity of *A. lwoffii* MA1, we conducted ATR-FTIR analysis on *A. lwoffii* MA1 before and after exposure to Cd. After the addition of Cd, the characteristic bending and stretching vibrations in the spectrum of *A. lwoffii* MA1 exhibited significant changes, indicating the formation of Cd-containing complexes (Figure 6f). Moreover, it was discovered that several peaks represented the primary functional groups. The wide peak near 3,300 cm^−1^ likely resulted from the stretching of amino groups (–NH2) and the strong polarity of hydroxyl groups (–OH). The peaks near 2,920 cm^−1^ and 2,960 cm^−1^ presented the stretch of alkane groups (–CH). The peak around 1,650 cm^−1^ was associated with the stretching of carboxylic groups (–COOH), and the band at about 1,540 cm^−1^ corresponded to N–H bending in proteins. The peaks at approximately 1,220 cm^−1^ and 1,450 cm^−1^ were mainly attributed to phosphate diesters and the symmetrical tensile vibration of C–O in the COO-NH3 of amide III groups. Particularly, the bands between 1,220 cm^−1^ and 8,000 cm^−1^ are dominated by C–OH and P–O stretching vibrations in polysaccharides or nucleic acids. From this, we infer that these functional groups likely provide various binding sites, facilitating the binding and removal of Cd^2+^.

### 3.6 Mechanism of Cd adsorption by membrane proteins

The side chains of amino acid residues in many membrane proteins are exposed on the cell surface, and it is likely that these groups are involved in the Cd adsorption process. To verify the role of key functional groups during Cd adsorption, three specific groups (amino, carboxyl, and phosphate) were selected for investigation. Selective chemical masking approach were employed to sequentially block each target functional group while preserving the others, thereby elucidating the individual contributions of these groups to Cd^2+^ binding on the surface of *A. lwoffii* MA1. Masking three types of surface groups reduces the ability of *A. lwoffii* MA1 to absorb infrared radiation (Figure 7a). Masking amino groups caused an extremely significant 50.46% reduction in the Cd adsorption rate, whereas masking carboxyl groups resulted in only a 4.42% decrease (Figure 7b). However, no difference was detected in the Cd^2+^ adsorption rate after masking phosphate groups compared to untreated bacterial cells, indicating that the phosphate groups do not play a significant role in the binding of *A. lwoffii* MA1 to Cd^2+^. As fundamental groups in membrane proteins, amino and carboxyl groups are essential for Cd adsorption by *A. lwoffii* MA1, underscoring the central role of membrane proteins in heavy metal biosorption.

**Figure 7:**
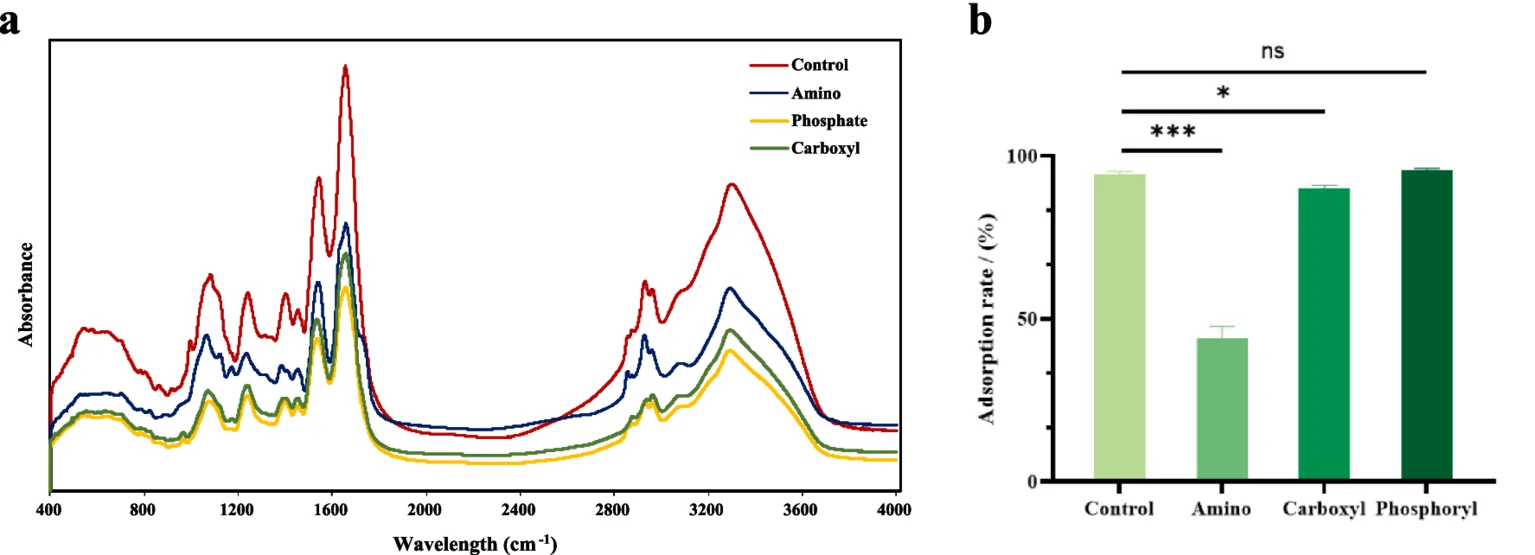
**(a)** ATR-FTIR and **(b)** Adsorption rate of *A. lwoffii* MA1 after masking the surface groups.

Membrane proteins were extracted from *A. lwoffii* MA1 and subsequently purified usingaffinity chromatography. The three protein bands observed on SDS-PAGE were analyzed by RPLC-MS peptide sequencing. The identity of a protein is determined by analyzing the masses of peptide fragments obtained from enzymatic digestion and comparing them against a database (Table 1). The putative membrane protein, TonB-dependent receptor, encodes a polypeptide of 686 amino acid residues, with a predicted molecular mass of 75.79 kDa. Multidrug efflux RND transporter AdeIJK encodes a polypeptide of 488 amino acid residues, with a predicted molecular mass of 53.15 kDa. Outer membrane protein assembly factor BamD encodes a polypeptide of 318 amino acid residues, with a predicted molecular mass of 36.28 kDa. They possess the capacity to adsorb Cd^2+^, implicating these membrane proteins in heavy metal interaction or detoxification mechanisms.

**Table 1:** Membrane proteins list.

Protein	Accession	Peptide spectrum matches	Coverage (%)	Unique peptides	MW (kDa)	Identified proteins
1	A0AAJ4P2D3	105	55.90	38	75.79	TonB-dependent receptor
2	A0AAJ4TUC1	112	62.50	26	53.15	Multidrug efflux RND transporter AdeIJK
3	A0AAJ3AI62	84	72.27	29	36.28	Outer membrane protein assembly factor BamD

## 4 Discussion

The establishment of plant species depends on symbiotic relationships with endophytic microorganisms, including both fungi and bacteria, which are tolerant to various environmental stresses (Rodriguez et al., 2008). Endophytic bacteria present in seeds can quickly colonize the new generation of plants because they reside within the host plants, where they face little competition for space and nutrients from external microbes and have adapted to live within plant tissues (Klironomos, 2002). Ringelberg et al., considered seeds as the main contributor to mature wheatgrass endophytes because they recovered the same genus from seeds and mature plant tissues (Ringelberg et al., 2012). In soybeans, it has been demonstrated that seed-borne bacteria can multiply and persist in seedlings, with nearly all bacterial genera isolated from seeds can also be isolated from the roots of these plants (Zhang et al., 2024). It can be seen that seeds are an important source of endophytic bacteria (Matsumoto et al., 2021). One of the major challenges in studying endophytic microorganisms is that epiphytic propagules, which are often related to the plant surface, can penetrate the superficial epithelial layer without actually becoming endophytes. To eliminate interference from both viable and nonviable propagules or epiphytes, surface sterilization was employed, allowing for the isolation of true endophytic microorganisms. Zhou et al., screened Cd-resistant endophytic strains from rice seeds and found that all genera found in rice seeds were also detected in other parts of the rice plant (Zhou et al., 2020). In this study, several endophytic strains were isolated after removing surface microorganisms from spinach seeds, and a highly Cd-tolerant endophytic bacterium, *A. lwoffii* MA1, was identified, exhibiting strong Cd adsorption capacity and membrane adsorption effect.

Cd is considered a nonessential element in plants, and its toxicity leads to general growth abnormalities and inhibition in several plant species. Exposure to toxic levels of Cd hinders seed germination, suppresses plant growth and production, disrupts seedling physiological processes, and reduces agricultural productivity (Raza et al., 2020). After prolonged exposure to Cd, roots undergo necrosis, decomposition, and mucilage production, which reduces the elongation of plant roots and buds, leading to leaf rolling and chlorosis (Abbas et al., 2018). The total leaf area and dry weight also decreased significantly in various plant components. The pot experiment showed that Cd does cause these damages to the growth of spinach. Additionally, the enrichment of *A. lwoffii* MA1 increased the Cd content in spinach, which corresponded to a greater degree of damage. The retardation of plant growth under Cd stress may be attributed to decreased nutrient and water adsorption, impaired respiration, reduced nitrogen, and carbon assimilation, as well as diminished antioxidant activity (Rizwan et al., 2017). The toxicity of Cd also reduces plant mineral adsorption and photosynthesis, which reduces crop yield and quality (Rizwan et al., 2016). High concentrations of Cd have been shown to inhibit the growth of cells and entire plants, especially for maize, alfalfa, wheat, soybean, and spinach (Rizwan et al., 2016).

The presence and accumulation of heavy metals in the edible portions of vegetables typically begin with accumulation in the roots, followed by adsorption and subsequent translocation to the aerial portions (Amin et al., 2013). Therefore, when Cd is present in the soil, it can accumulate in the edible parts of spinach, giving rise to food safety issues. Past experiments have shown that microbial flora (i.e., fungi, bacteria, and algae) can help eliminate both organic and metal contaminants (Parthipan et al., 2017). For example, *Bacillus* can effectively remove toxic pollutants such as lead, nickel, iron, copper, chromium, and Cd from contaminated agricultural soil or industrial wastewater (Radziemska et al., 2017). However, it was shown in this study that endophytic microorganisms are closely related to the content of Cd adsorbed in spinach. As shown in Figure 6, the content of *A. lwoffii* MA1 and Cd in normal seeds planted in normal soil is the lowest. When normal seeds are planted in high-Cd soil, both the content of *A. lwoffii* MA1 and Cd increases. For seeds enriched with endophytic microorganisms and planted in high-Cd soil, the content of *A. lwoffii* MA1 and Cd increase even further. This finding indicates that endophytic bacterial increase in content can facilitate the accumulation of heavy metals in the edible portions of vegetables, and the spinach seeds reinforced with endophytic bacteria can absorb large amounts of Cd^2+^. Therefore, Cd uptake by plants can be reduced by decreasing the proportion of *A. lwoffii* MA1 in spinach seeds, thereby enhancing the safety of vegetable consumption. Additionally, *A. lwoffii* MA1 has the ability to adsorb Cd^2+^ and detoxify heavy metals, suggesting its potential application in fields such as soil bioremediation.

Depending on the binding sites of the cellular molecular structures and the affinity of various metal ions or ionic forms, certain bacteria can either actively or passively acquire the ability to absorb relatively high quantities of heavy metals from liquids. There are three main steps involved in Cd accumulation within the cells. First, Cd^2+^ are rapidly fixed on the cell surface through the synergistic interaction of electrostatic attraction and coordination with functional group. Then, a part of Cd^2+^ is transported into the cytoplasm, where they bind to certain intracellular substances. Finally, with increasing intracellular Cd content, cells activate efflux system to maintain intracellular Cd homeostasis. The amino, carboxyl, and phosphonate functional groups, which play key roles in heavy metal adsorption, are distributed on the cell surface of bacteria, serving as the initial sites of metal ion interaction (Nanda et al., 2019). With high concentrations of heavy metals entering the cell membrane, the production of ROS can rapidly attack biomolecules, such as proteins, amino acids, nucleic acids, and lipids (Pospíšil et al., 2019). Hence, Cd^2+^ binding to multiple chemical groups in the cell membrane can inhibit its entry into the cell, thereby reducing the toxicity of Cd to the bacterial cytoplasm (Wang et al., 2022).

Therefore, when the hydrogen ions on the cell surface amino groups are replaced by methyl groups, the amino groups lose their ability to react with metal ions, leading to a significant decrease in the adsorption rate of Cd^2+^ by *A. lwoffii* MA1. Additionally, the cell surface contains a large number of negatively charged carboxyl groups. Through ion exchange and electrostatic interactions, these groups can absorb divalent ions such as Cd^2+^ while releasing hydrogen ions (Chakravarty and Banerjee, 2012). However, when the carboxyl groups on the cell surface are methylated and lose their ion exchange capacity, the adsorption rate of Cd^2+^ on *A. lwoffii* MA1 cells is reduced. In contrast, phosphate groups have little to no effect on Cd^2+^ adsorption, which may be due to differences in the surface functional group characteristics among bacterial strains. Thus, *A. lwoffii* MA1 participates in Cd^2+^ adsorption by forming complexes with organic functional groups such as amino and carboxyl groups on the cell surface. Moreover, extracellular polymeric substances (EPS), primarily composed of polysaccharides, proteins, nucleic acids, and lipids, play a crucial role in protecting bacteria from heavy metal toxicity (Nocelli et al., 2016). However, when Cd^2+^ concentrations reach a certain threshold, Cd^2+^ would penetrate the cell membrane and enter the cytoplasm. Qualitative analysis of Cd signals on both the *A. lwoffii* MA1 cell surface and intracellular space indicates that Cd^2+^ not only adsorbs to the cell membrane but also crosses it into the cytosol. To protect itself from damage, *A. lwoffii* MA1 activates an efflux mechanism to expel Cd^2+^ from the cytoplasm to the extracellular space, resulting in significant accumulation of Cd^2+^ on the cell membrane, which is consistent with the findings of Junhui et al. (Zhang et al., 2019).

The cell membrane is primarily composed of phospholipids, which serve as its fundamental structural units, with proteins either embedded within or attached to the phospholipid bilayer. These membrane proteins play vital roles in various biological processes, including molecular transmembrane transport, energy conversion, and chemical signal transduction. Notably, polar amino acid residues on the surfaces of membrane protein surfaces (such as the carboxyl group of glutamate, the sulfhydryl group of cysteine, and the imidazole group of histidine) can directly capture Cd^2+^ through electrostatic interactions or coordination bonds (Lu and Fu, 2007). This suggests that membrane proteins likely play a significant role in Cd^2+^ adsorption. In this study, we identified three Cd-adsorbing membrane proteins from the cell membrane of *A. lwoffii* MA1. The TonB-dependent receptor is a crucial class of outer membrane proteins in Gram-negative bacteria, characterized by a *β*-barrel transmembrane domain that forms substrate-translocating channels, along with extracellular loops containing substrate-specific binding pockets (Ferguson and Deisenhofer, 2002). Importantly, certain TonB-dependent receptors exhibit remarkable metal-binding capabilities, participating in microbial Cd accumulation through dual mechanisms: direct adsorption of free Cd^2+^ ions via coordination with extracellular binding sites, and/or recognition of Cd-organic complexes (Wan et al., 2022). The multidrug efflux RND transporter AdeIJK is a multidrug efflux pump found in Gram-negative bacteria and belongs to the Resistance-Nodulation-Division (RND) family of transporter proteins. Its primary function is to mediate multidrug resistance by actively pumping out a wide range of antibiotics and chemotherapeutic agents, thereby enabling bacterial resistance to these drugs (Damier-Piolle et al., 2008). Additionally, it may also participate in the efflux of other harmful substances, aiding bacterial adaptation to environmental stress. However, recent studies have found that RND family transporters (including AdeIJK) may indirectly contribute to the adsorption and detoxification of Cd^2+^ through mechanisms such as directly effluxing heavy metal ions, regulating biofilm formation, and indirectly promoting adsorption (Kornelsen and Kumar, 2021). The outer membrane protein assembly factor BamD is a crucial component of the β-barrel outer membrane protein assembly BAM complex, and it is responsible for regulating the proper folding and insertion of outer membrane proteins (Volokhina et al., 2009). BamD may also be involved in the adsorption of Cd^2+^ through mechanisms such as regulating the assembly of outer membrane proteins related to heavy metal transport, and maintaining the integrity of the outer membrane (Noinaj et al., 2013).

Based on the above analysis, we propose a possible mechanism by which endophytic microorganisms *A. lwoffii* MA1 increases Cd adsorption. Under stress induced by Cd, *A. lwoffii* MA1 initiates biosorption and captures the positively charged Cd^2+^ through negatively charged cellular groups on its surface, such as amino and carboxyl groups. In addition, three kinds of membrane proteins in the cell membrane play a major role in the adsorption process, including TonB-dependent receptor, RND transporter AdeIJK, and Outer membrane protein assembly factor BamD. However, several limitations of this study should be acknowledged. This study has several limitations. First, the experiments were conducted on potted plants under controlled greenhouse conditions, which may not fully represent the complex environmental factors influencing cadmium bioavailability and bacterial performance in the field, such as soil type, pH, organic matter content, competing cations, and interactions among microbial communities. Second, our assessment was limited to a single strain (*A. lwoffii* MA1), leaving it unclear whether co-inoculation with other endophytic strains or native soil microbes would enhance, inhibit, or have no effect on cadmium accumulation in spinach. Third, the increase in cadmium content in spinach leaves demonstrated that this strain can influence cadmium accumulation; however, it also raises food safety concerns. Our study was limited to observing the increase in cadmium content in spinach, and further research should focus on using endophytic microbes to reduce cadmium levels. Future studies should include field-scale trials across various soil types and environmental conditions, evaluate the effects of different strain combinations, and investigate strategies to limit cadmium translocation to edible tissues while preserving the beneficial effects on plant growth.

## Data Availability

The mass spectrometry proteomics data have been deposited to the ProteomeXchange Consortium (https://proteomecentral.proteomexchange.org) via the iProX partner repository with the dataset identifier PXD083107.
