# Supplementary material for: Endophytic microbe Acinetobacter lwoffii MA1 alters the cadmium content in Spinacia oleracea through membrane proteins-mediated adsorption
Source: Front Microbiol. 2026 Sep 16;17:1922017. doi: 10.3389/fmicb.2026.1922017 (PMC13624141; doi:10.3389/fmicb.2026.1922017)
Supplement: Supplementary file 1 [file Table_1.DOCX]

Table S1 MNP site of InternalGene and *Acinetobacter lwoffii*

| Loci | primer_F | primer_R | Species |
| --- | --- | --- | --- |
| MNP01 | CCCTGATAGAATAAGTAGGCACCAA | TACCATATCAAATGTGTTCCCGGAT | InternalGene |
| MNP02 | TTTGCTTTCGCTCAACTTTGTAGAT | TGCATGTTTTTCTTTTGTGGAACTG | InternalGene |
| MNP03 | CAGCAAATGAAGACCTGGTATTGAA | GTCATAGTCATCAGCGCAAATTACA | InternalGene |
| MNP04 | TGCACCCCTAAAATTTATGGAACTG | ATAATGTTCAGCCACTTTTGTCCTC | InternalGene |
| MNP05 | tgcacGTAATTGGTTGTAGTGTAAC | AAACTGCTTGCATATATTTTGCAGC | InternalGene |
| MNP06 | ATAATGTAAATCAAGCAGAAAAGCTG | CGCCCTTCATAAACCTTATCTGTTC | Acinetobacter_lwoffii |
| MNP07 | ACTTTATCTTGGTGTCACTTAAGTG | TCATGACCTCTAAAGTGATTACCGT | Acinetobacter_lwoffii |
| MNP08 | TAATTTCGTGGTGTGAGCCAAATG | GCCTGATGGACTTGCTGATATTTTT | Acinetobacter_lwoffii |
| MNP09 | CCATATCCCACATACCACCATAACT | GAATCGTCCACAGTTTGAAGTTGAA | Acinetobacter_lwoffii |
| MNP10 | TGTTCTTCTTTAACATTTGCTGAACG | CGGATTTTAAACCGGATTCTGACC | Acinetobacter_lwoffii |
| MNP11 | AATACCGACAGGAATTTGCGTATTC | TCATAAAGCTGGTTTTCGCATACAG | Acinetobacter_lwoffii |
| MNP12 | ATGATTGTTTCCTATCAATTCGCCC | ACTGAATGGTGGTTTCAACTTTGAA | Acinetobacter_lwoffii |
| MNP13 | CTTGCACGAAATTACCAGTCTATGA | TCCTACGAGAAAAATTATTGTATCGTGA | Acinetobacter_lwoffii |
| MNP14 | ATCAACCACTTCCGTTTTCATGATC | CACTGGAAGACCGTTTGATTAAACA | Acinetobacter_lwoffii |
| MNP15 | AGAGCAGGAAACTCCCAATCATAAT | TTCCATGGATGTTTTAGAGTGCATT | Acinetobacter_lwoffii |
| MNP16 | CCTGGTTTTTAAGAATCCACTGGTC | CACGTGCTGCTTTTGACAAAAA | Acinetobacter_lwoffii |
| MNP17 | GTCGCGGAAGGCTATATTGATTATC | TTGTTGTAATTGTTCAGGATCGGTC | Acinetobacter_lwoffii |
| MNP18 | GACAAATTGAACAGAGATCGAGCAT | ATCACCAGACTTTTGGCATTTTTCT | Acinetobacter_lwoffii |
| MNP19 | CATGGGACGACTCCAAAGAAAAATA | TTGTTCTTGCTGAATAACAAGGCAT | Acinetobacter_lwoffii |
| MNP20 | ATACAGGACTGGTCGATCTAATCAC | AATGTTTCAATACCTTCTGCACTGG | Acinetobacter_lwoffii |
| MNP21 | ATTTTCGTCATAGGGTCGATTTTCG | CCCTGCACGATTTCGAGTTTATTAT | Acinetobacter_lwoffii |
| MNP22 | CGCCCAGTAAAACAAACATACTGAT | AAGATAGGCTGCTGTTAAAACCAAC | Acinetobacter_lwoffii |
| MNP23 | TGTATTTTCTGCGGTATGTGTGAAG | CCACATCATGGTAATAGACTCCGTA | Acinetobacter_lwoffii |
| MNP24 | CTTTGCGTGCCCAGATATTATCTAC | CGATTTAAGCCTGCAACTATTACGT | Acinetobacter_lwoffii |
| MNP25 | TCGACCTTAGTGAGAATCTTAAATTC | TGTTGATATGATCGCAGTAGGTCAT | Acinetobacter_lwoffii |
