# Supplementary material for: Endophytic microbe Acinetobacter lwoffii MA1 alters the cadmium content in Spinacia oleracea through membrane proteins-mediated adsorption
Source: Front Microbiol. 2026 Sep 16;17:1922017. doi: 10.3389/fmicb.2026.1922017 (PMC13624141; doi:10.3389/fmicb.2026.1922017)
Supplement: Supplementary file 2 [file Table_2.DOCX]

Table S2 16S rRNA gene sequence of Acinetobacter lwoffii MA1

GGGAATGGCGTCTGAGCTTGATATCGAATTCGCGTGTCGCCCTTCGGCTACCTTGTTACGACTTCACCCCAGTCATCTGCCACACCGTGGTAAGCGTCCTCCTTGCGGTTAGGCTACCTACTTCTGGTGCGACAAACTCCCATGGTGTGACGGGCGGTGTGTACAAGGCCCGGGAACGTATTCACCGCGGCATTCTGATCCGCGATTACTAGCGATTCCGACTTCATGGAGTCGAGTTGCAGACTCCAATCCGGACTACGATCGGCTTTTTGAGATTAGCATCCTCTCGCGAGGTAGCAACCCTTTGTACCGACCATTGTAGCACGTGTGTAGCCCTGGTCGTAAGGGCCATGATGACTTGACGTCGTCCCCGCCTTCCTCCAGTTTGTCACTGGCAGTATCCTTAAAGTTCCCGGCTTAACCCGCTGGCAAATAAGGAAAAGGGTTGCGCTCGTTGCGGGACTTAACCCAACATCTCACGACACGAGCTGACGACAGCCATGCAGCACCTGTATGTAAGTTCCCGAAGGCACCAATCCATCTCTGGAAAGTTCTTACTATGTCAAGACCAGGTAAGGTTCTTCGCGTTGCATCGAATTAAACCACATGCTCCACCGCTTGTGCGGGCCCCCGTCAATTCATTTGAGTTTTAGTCTTGCGACCGTACTCCCCAGGCGGTCTACTTATCGCGTTAGCTGCGCCACTAAAGCCTCAAAGGCCCCAACGGCTAGTAGACATCGTTTACGGCATGGACTACCAGGGTATCTAATCCTGTTTGCTCCCCATGCTTTCGCACCTCAGTGTCAGTATTAGGCCAGATGGCTGCCTTCGCCATCGGTATTCCTCCAGATCTCTACGCATTTCACCGCTACACCTGGAATTCTACCATCCTCTCCCATACTCTAGCCAACCAGTATCGAATGCAATTCCCAAGTTAAGCTCGGGGATTTCACATTTGACTTAATTGGCCACCTACGCGCGCTTTACGCCCAGTAAATCCGATACGCTTGCACCCTCTGTATTACCGCGCTGCTGCACAGAGTAGCGTGCTTATTTCTGCGAGGTAACGTCCACTATTCCAAGAAGTATTAAATCTCTCGGGTAGCTC
